# Rapid centralised randomisation in emergency setting trials using a smartphone

**DOI:** 10.1007/s00431-022-04475-y

**Published:** 2022-05-17

**Authors:** Shiraz Badurdeen, Kate A. Hodgson, Georgia A. Santomartino, Luke Stevens, Susan Donath, Calum T. Roberts, Brett J. Manley, Graeme R. Polglase, Stuart B. Hooper, Peter G. Davis, Douglas A. Blank

**Affiliations:** 1 Newborn Research Centre, The Royal Women’s Hospital, 20 Flemington Rd, Parkville, VIC 3052 Australia; 2 The Ritchie Centre, Hudson Institute of Medical Research, Clayton, VIC Australia; 3 Department of Obstetrics and Gynaecology, The University of Melbourne, Melbourne, VIC Australia; 4 Clinical Epidemiology and Biostatistics Unit, Murdoch Children’s Research Institute, Parkville, VIC Australia; 5 Department of Paediatrics, Monash University, Wellington Rd, Clayton, VIC Australia; 6 Monash Newborn, Monash Children’s Hospital, Clayton, VIC Australia; 7 Clinical Sciences Research, Murdoch Children’s Research Institute, Melbourne, Australia; 8 Department of Obstetrics and Gynaecology, Monash University, Wellington Rd, Clayton, VIC Australia

**Keywords:** Neonatal, Randomised controlled trial

## Abstract

Randomised trials in emergency settings must quickly confirm eligibility and allocate participants to an intervention group without delaying treatment. We report rapid randomisation during two neonatal resuscitation trials using the non-commercial REDCap platform accessed via smartphone. This simple, reliable method has wide applicability for trials in emergency settings.

**Table:** 

**What is Known:** *• Randomised trials in emergency settings need to rapidly allocate participants to an intervention group.* *• *This process should not delay treatment.	
**What is New:** *• This non-commercial, smartphone-accessible application enabled rapid, accurate randomisation at the bedside.* *• This has broad applicability for emergency setting trials.*

**Supplementary Information:**

The online version contains supplementary material available at 10.1007/s00431-022-04475-y.

## Introduction

Clinicians and researchers conducting randomised trials in emergency settings face the challenge of needing to quickly confirm eligibility and allocate participants to an intervention group without delaying treatment. The urgency is particularly acute in delivery room trials of neonatal resuscitation, which require participant allocation and application of the intervention within seconds of determining eligibility. As a result, trials have historically used methods that are at risk of bias such as sealed envelopes, allocation prior to eligibility confirmation, or quasi-randomisation [1–3]. To ensure validity, the allocation process should be methodologically rigorous, minimise treatment delay, and streamline trial procedures. We report our experience with performing rapid randomisation during neonatal resuscitation using the smartphone-accessible, non-commercial REDCap platform — a database management system [4]. The aim of this study was to evaluate the use of the online randomisation tool for two delivery room randomised controlled trials.

## Materials and methods

We used randomisation via REDCap for two unblinded, parallel-arm neonatal resuscitation trials conducted at The Royal Women’s Hospital and Monash Medical Centre in Melbourne, Australia. The Human Research Ethics Committees at each site approved both trials. In the Baby-Directed Umbilical Cord Cutting (BabyDUCC) trial (ACTRN12618000621213) [5], eligible infants ≥ 32^+0^ weeks’ gestation who required resuscitation within 1 min of birth were randomised to receive respiratory support in the maternal space with the umbilical cord still intact (intervention) or after clamping the umbilical cord and transferring the infant to the warming bed. In the Stabilisation with Nasal High Flow for Intubation of Neonates (SHINE) trial (ACTRN12618001498280) [6], infants undergoing endotracheal intubation were randomised to receive nasal high flow during the intubation attempt, or to standard care (no nasal high flow). The subset of infants in the SHINE trial intubated in the delivery room during neonatal resuscitation was included in the current analysis. Infants intubated in the neonatal unit were excluded as rapid assessment of eligibility and randomisation was not required [5, 6].

**Fig. 1:**
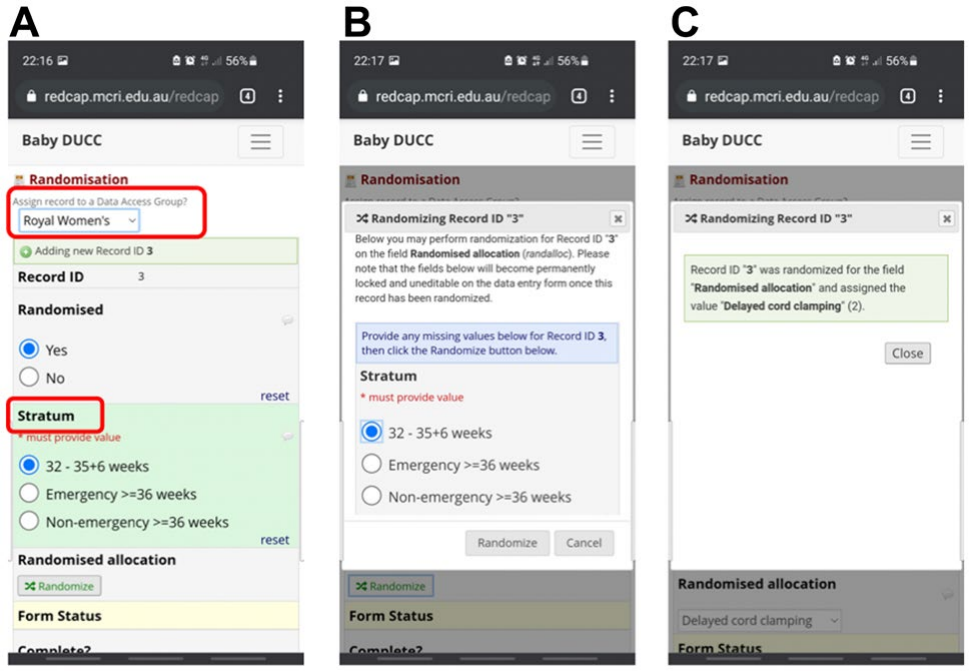
Screenshots of the randomisation process. A weblink to the “New Randomisation” (**A**) is accessed through a weblink shortcut saved on the site investigator’s smartphone home screen. This page requires the user to log in. Strata are selected as shown in the red boxes. Once participant eligibility is confirmed, the investigator presses the “Randomise” button (step 1). A window (**B**) appears to confirm the strata, and the decision to randomise is confirmed by pressing the “Randomise” button again (step 2). The group allocation is then revealed (**C**) and a record simultaneously created for the participant. The allocation is permanently locked into the participant’s REDCap data entry form and cannot be edited

A readily accessible, rapid randomisation tool was required to allow treatment allocation at the participant’s bedside without delaying essential resuscitation care. For each trial, a pre-generated randomisation schedule was uploaded to the REDCap randomisation module by an independent statistician. Site investigators accessed the “New Randomisation” page via a password-protected mobile phone weblink that allowed advance selection of strata. The randomisation tool could be accessed by multiple investigators using their unique REDCap login profile on their smartphones, providing additional automatic stratification by site (“Data Access Group”). Enrolment and randomisation of patients could therefore occur simultaneously at the two sites. Following birth, eligibility was assessed. If the infant required resuscitation within 1 min of birth (BabyDUCC trial) or endotracheal intubation (SHINE trial), the researcher or assistant would reveal the allocation via a two-step randomise-and-confirm process (Fig. 1). This involved pressing the “randomise” button on the initial screen page (step 1) and again on a confirmation pop-up window (step 2). For caesarean births in the BabyDUCC trial, a midwife not involved in the study randomised the infant and revealed the allocation as the researcher was scrubbed in the operating field.

Both trials incorporated video and audio recordings (GoPro, USA) of resuscitation that captured the randomisation events (Supplementary Fig. 1). A single researcher who was not present at the births (GS) reviewed the recordings to determine the time between the decision to randomise and the disclosure of group allocation. Decision to randomise was defined as the time when the researcher confirmed eligibility in conjunction with the clinical team. In the BabyDUCC trial, this was a call to randomise the infant following an assessment that the infant required resuscitation to establish breathing. In the SHINE trial, the decision to randomise was when the clinical team confirmed to the researcher the intention to intubate the infant. Allocation revelation was defined as the time when the study allocation was verbalised to the clinical team. Where the allocation was not immediately verbalised, for instance when the clinical team was busy with other resuscitation tasks, allocation revelation was defined as the time when the allocation appeared on the smartphone device as captured by the recording.

Supplementary Fig. 1. Infants enrolled in the Baby-Directed Umbilical Cord Cutting (BabyDUCC) trial and the Stabilisation with Nasal High Flow for Intubation of Neonates (SHINE) trial with available data for evaluation of the randomisation tool. 

*The number of births attended for the SHINE trial was unavailable, but no infants were accidentally randomised prior to confirmation of eligibility.

## Results

In the BabyDUCC trial (*N* = 123, Supplementary Fig. 1), the decision to randomise occurred at a mean (SD) of 25.2 (11.9) seconds after birth. Group allocation was revealed 5.9 (2.9) seconds after the decision to randomise. Two randomisation errors occurred: in each case, an assistant had progressed through the initial “Randomise” step (step 1) followed by accidental confirmation (step 2) before birth. We subsequently enforced the planned approach of waiting until after birth to progress past step 1. Following enforcement of the two-step randomise-and-confirm process after birth and assessment of eligibility, no further errors occurred (*N* = 94).

In the SHINE trial (*N* = 68, Supplementary Fig. 1), the two-step randomise-and-confirm process after confirmation of eligibility was employed throughout. Group allocation was revealed at a mean (SD) of 6.8 (4.9) seconds after the decision to randomise. One post-randomisation exclusion occurred when a randomisation was appropriately confirmed in a patient that was initially planned for endotracheal intubation but subsequently was not intubated.

All eligible infants were successfully randomised in each trial. There were no instances of technical difficulties related to internet access or REDCap server downtime. The supplementary online videos provide representative examples of the randomisation tool being used (Supplementary videos 1 & 2).

## Discussion

We have described our experience with the REDCap randomisation tool accessed using a smartphone browser. The tool was reliable, easy to use, and facilitated group assignment within seconds of confirming patient eligibility. We recommend adhering to the two-step randomise-and-confirm process to avoid accidental randomisation.

Other scenarios where the provider must quickly confirm eligibility and allocate participants to an intervention group without delaying treatment include trials of airway interventions and out-of-hospital cardiac arrest. Recent major trials in these fields used sealed envelopes [7], randomisation of the provider [8], opening of a pre-randomised trial drug kit [9], and randomisation of the day of the week [10]. The method we report here allows methodologically rigorous randomisation of the individual participant following confirmation of eligibility. A previously reported mobile app for rapid randomisation [11] is no longer available (personal communication) and we are not aware of other similar platforms.

The advantages of the method we used include centralised, stratified randomisation that can be performed at the bedside using any internet- or mobile data–enabled smartphone. Multiple investigators can use the tool across different recruitment sites including locations worldwide. Allocation concealment is rigorous and there is automatic lock of the group allocation within the study database. The system also supports blinding of group allocation for trials where this is required. To enable others to replicate this method, we include the REDCap ODM XML “metadata-only” file for the BabyDUCC trial (Supplemental File) that can be easily modified to suit the individual trial’s requirements. Although not used in our trials, dynamic randomisation by minimisation can be implemented in REDCap using a custom module developed by one of the study authors (LS). This tool has broad applicability for trials in different emergency settings.

## Supplementary Information

Below is the link to the electronic supplementary material.

Supplementary file1: RandTool_SupplVid_1 (MP4 67 MB)

Supplementary file2: RandTool_SupplVid_2 (MP4 56 MB)

Supplementary file3 (PDF 39 KB)

Supplementary file4 (ZIP 2 KB)

## Data Availability

Data will be available upon reasonable request following completion and publication of each of the main trials.
