# Supplementary figures and images for: Rapid centralised randomisation in emergency setting trials using a smartphone
Source: Eur J Pediatr. 2022 May 17;181(8):3207–10. doi: 10.1007/s00431-022-04475-y (PMC9352638; doi:10.1007/s00431-022-04475-y)

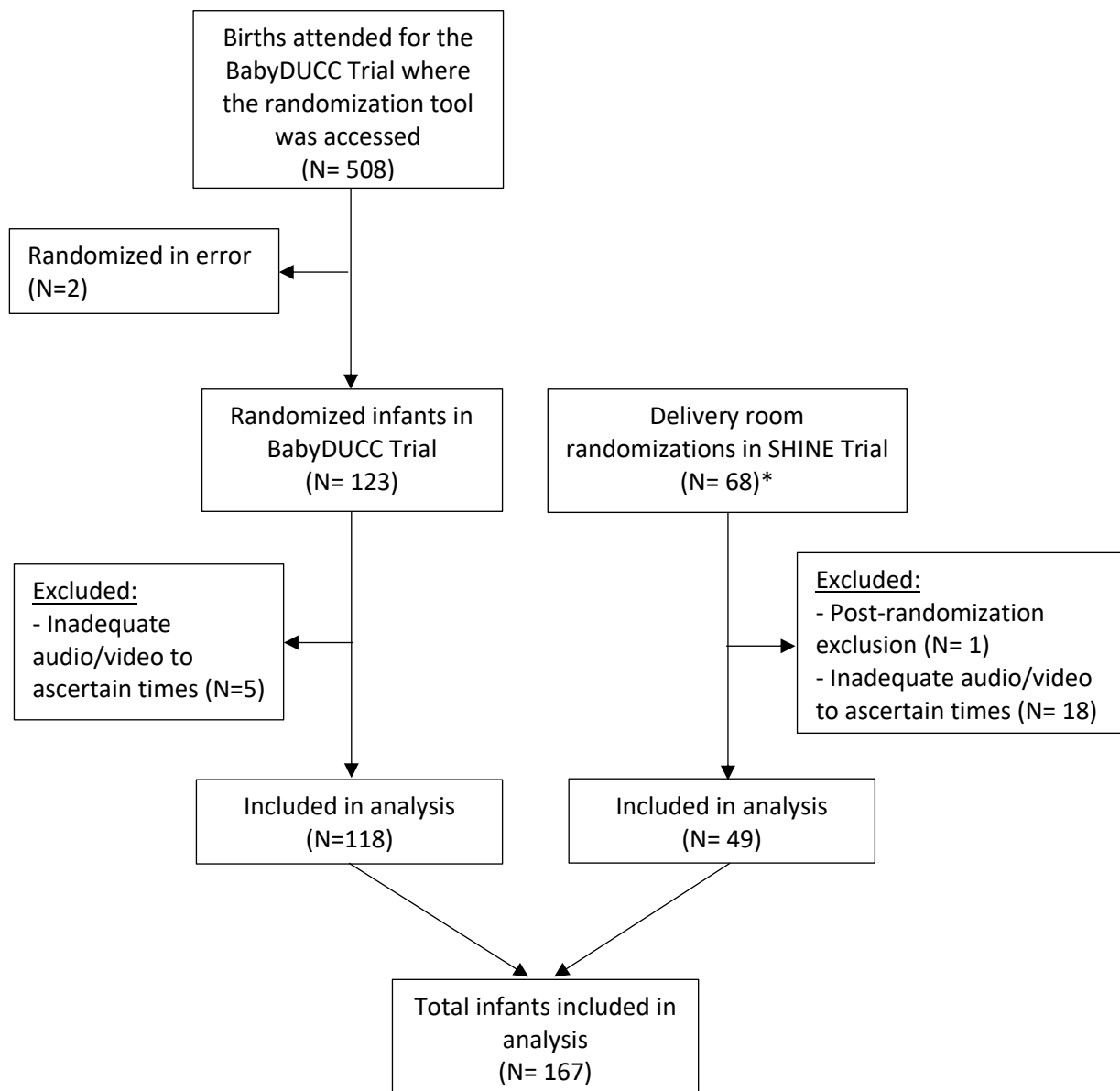

Supplement: Supplementary file 3 — Supplementary file3 (PDF 39 KB) [file 431_2022_4475_MOESM3_ESM.pdf]
